# Epigenetic Changes Associated With Exposure to Glyphosate-Based Herbicides in Mammals

**DOI:** 10.3389/fendo.2021.671991

**Published:** 2021-05-21

**Authors:** María Florencia Rossetti, Guillermina Canesini, Virginia Lorenz, María Mercedes Milesi, Jorgelina Varayoud, Jorge Guillermo Ramos

**Affiliations:** ^1^ Instituto de Salud y Ambiente del Litoral (ISAL), Universidad Nacional del Litoral (UNL)- Consejo Nacional de Investigaciones Científicas y Técnicas (CONICET), Facultad de Bioquímica y Ciencias Biológicas, Universidad Nacional del Litoral, Santa Fe, Argentina; ^2^ Departamento de Bioquímica Clínica y Cuantitativa, Facultad de Bioquímica y Ciencias Biológicas, Universidad Nacional del Litoral, Santa Fe, Argentina; ^3^ Cátedra de Patología Humana, Facultad de Bioquímica y Ciencias Biológicas, Universidad Nacional del Litoral, Santa Fe, Argentina; ^4^ Cátedra de Fisiología Humana, Facultad de Bioquímica y Ciencias Biológicas, Universidad Nacional del Litoral, Santa Fe, Argentina

**Keywords:** glyphosate, amino methylphosphonic acid (AMPA), epigenetic, DNA methylation, histone modifications, miRNA

## Abstract

Glyphosate is a phosphonomethyl amino acid derivative present in a number of non-selective and systemic herbicides. During the last years the use of glyphosate-based herbicide (GBH) has been increasing exponentially around the world, including Argentina. This fact added to the detection of glyphosate, and its main metabolite, amino methylphosphonic acid (AMPA), in environmental matrices such as soil, sediments, and food, has generated great concern about its risks for humans, animals, and environment. During the last years, there were controversy and intense debate regarding the toxicological effects of these compounds associated with the endocrine system, cancer, reproduction, and development. The mechanisms of action of GBH and their metabolites are still under investigation, although recent findings have shown that they could comprise epigenetic modifications. These are reversible mechanisms linked to tissue-specific silencing of gene expression, genomic imprinting, and tumor growth. Particularly, glyphosate, GBH, and AMPA have been reported to produce changes in global DNA methylation, methylation of specific genes, histone modification, and differential expression of non-coding RNAs in human cells and rodents. Importantly, the epigenome could be heritable and could lead to disease long after the exposure has ended. This mini-review summarizes the epigenetic changes produced by glyphosate, GBHs, and AMPA in humans and rodents and proposes it as a potential mechanism of action through which these chemical compounds could alter body functions.

## Introduction

Epigenetics is defined as ‘molecular factors and processes around DNA that regulate genome activity, independent of DNA sequence, and are mitotically stable’ (1). Epigenetic modifications include DNA methylation, post-translational modifications of histones, and differential expression of non-coding RNAs. Epigenetic processes may be related to silencing/activating of gene expression, genomic imprinting, and pathology development (2–6). Moreover, epigenetic marks could be maintained over time and be transmitted transgenerationally in second, third, and fourth generations (7).

The epigenome is influenced by both genetic (*e.g.* single nucleotide polymorphisms) and environmental factors (8–10). In this sense, several studies indicate that pesticides can exert toxicity through epigenetic changes [reviewed in (11)]. Among environmental chemicals, glyphosate-based herbicides (GBHs) have been one of the most intensively used pollutants over the last two decades. The herbicide glyphosate, N-(phosphonomethyl) glycine, is a biocide with a broad-spectrum activity since its mode of action is by inhibiting the enzyme 5-enolpyruvylshikimate-3- phosphate synthase, involved in the biosynthesis of aromatic compounds in plants and microorganisms (12). Monitoring studies have evidenced the presence of glyphosate residues and its main metabolite, (aminomethyl) phosphonic acid (AMPA), in surface water, sediments, and soil (13–16), respirable dust emitted by agricultural soil (17), a variety of crops at harvest and processed food (18–20), human urine samples (21, 22), maternal and umbilical cord serum (23), and breast milk samples (24). The widespread presence of these compounds shows that there is a risk of environmental exposure and concern about their possible effects on the environment and human health.

Several studies have reported adverse effects of GBH and glyphosate exposure on female and male murine reproductive systems, at both low and environmentally relevant doses, including disruption of the hypothalamic–pituitary-axis (25), uterine and ovary abnormalities, pre- and post-implantation embryo losses [reviewed in (26)] and testicular lesions (27). Supporting these results, *in vitro* studies found alterations in sperm motility and mitochondrial functions in human sperm cells (28, 29), as well as increased death of TM4 Sertoli cells (30) and disruption of blood–testis barrier integrity (31). However, there have been controversy and debate regarding the toxicological effects of these compounds. While the International Agency for Research on Cancer (IARC) concluded in March 2015 that the herbicide and its formulated products are probably carcinogenic in humans (IARC Group 2A) (IARC 2015, https://www.iarc.fr); the European Food Safety Authority (EFSA) decided that ‘glyphosate is unlikely to pose a carcinogenic risk to humans’ (EFSA 2015) (32). In 2017, the Environmental Protection Agency (EPA) issued a Draft Human Risk Assessment for Glyphosate, which concluded that glyphosate is not likely to be carcinogenic in humans. In 2019, the EPA released a Glyphosate Proposed Interim Registration Review Decision for public comments and, in 2020, released the Interim Registration Review Decision. The EPA continues to find that there are no risks to public health when glyphosate is used in accordance with its current label (EPA 2020 https://www.epa.gov).

The mechanisms of action of GBH and their metabolites are still under investigation. It has been reported that they could comprise interference with Ca+ ion-channels and peptide/steroid hormone response [reviewed in (11)]. More recently, epigenetic mechanisms have been also proposed as possible mediator of the action of these compounds (**Table 1**). This mini-review summarizes the current evidence about glyphosate-, GBH- and AMPA-induced epigenetic modifications in humans and rodents and proposes them as potential mechanisms through which these compounds could alter body functions.

**Table 1:** Epigenetic modifications induced by glyphosate, glyphosate-based herbicides (GBH) and amino methylphosphonic acid (AMPA).

Compound	Modification	Tissue/Cell	Main Effects
Humans			
Glyphosate	DNA methylation (33)	PBMC	Decrease global 5mC percentage Increase methylation of p53 promoter
Glyphosate	DNA methylation (34)	PBMC	Decrease global 5mC percentage Change methylation pattern of p21 and p53 promoters Alter the expression genes involved in regulation of cell cycle (CCND1, p16, P21, and P53) and apoptosis (BCL2)
Glyphosate	DNA methylation (35)	MCF10A cells	DNA hypomethylation occurring *via* TET pathway Changes in methylation patterns of MTRNRL2 and DUX4 genes Exposure to glyphosate and miRNA 182-5p induced tumor development in 50% mice TET inhibitor prevents tumor formation in glyphosate-miR 182-5p-cells
AMPA and Glyphosate	DNA methylation and histone modification (36)	PBMC	Changes in the expression of DNMT1 and HDAC3 by glyphosate and AMPA Changes in the expression of DMNT3A by glyphosate
AMPA	DNA methylation (36)	PBMC	Decrease global 5mC percentage Change methylation pattern of p21 and p53 promoters Increase the expression of CCND1
Rodents			
GBH	DNA methylation (37)	Rat mammary gland	Alter mammary gland development Decrease ER*α* protein and mRNA expression (transcripts OS, O, OT, and E1) Changes in methylation patterns of ER*α* promoters in post-puberal animals
GBH	DNA methylation and histone modification (38)	Rat uterus	Increase ER*α* mRNA during pre-implantation period Increase the relative abundance of ER*α*-O transcript variant Alter methylation status and histone post-transductional modifications in the O promoter of ER*α* gene.
GBH	miRNA (39)	Mouse brain (PFC)	55 upregulated and 19 downregulated miRNAs Alter Wnt/*β*-catenin and Notch pathways
GBH	circRNA (40)	Mouse brain (Hip)	330 upregulated and 333 downregulated circRNAs
Glyphosate	DNA methylation (41)	Rat sperm	Increase pathologies in F2 and F3 (prostate, ovarian and kidney diseases, obesity, birth abnormalities, and tumor growth) DMRs in sperm
Glyphosate	DNA methylation and histone modification (42)	Rat sperm	DMRs and DHRs in F3 generation.

DHRs, Differential histone retention sites; DMRs, Differential DNA methylation sites; DNMT, DNA methyltransferase; HDAC, histone deacetylase; PBMCs, Peripheral Blood Mononuclear Cells; PFC, Prefrontal Cortex; Hip, Hippocampus.

## Epigenetic Mechanisms

DNA methylation is one of the most studied epigenetic modification. It occurs when DNA methyltransferases (DNMTs) transfer, in a reversible way, methyl groups from S-adenyl methionine to the fifth carbon of a cytosine residue, that is followed by a guanosine (CpG site), to form 5-methylcytosine (5mC) (43). The frequency of CpG sites is higher than expected in clusters known as CpG Island that are generally located in the promoter regions of genes. When these sites are methylated, transcription factors are not able to bind to the promoter regions, and the gene expression decreased. On the contrary, if these sites are demethylated, transcription is activated and gene expression is increased (9, 44).

Histone methylation could help to direct DNA methylation patterns, and DNA methylation seems to serve as a template for rebuilding histone modification patterns following DNA replication (45). Histone modification occurs on specific amino acid residues, changing the structure of chromatin and leading, together with the DNA methylation, to the activation or suppression of gene transcription (46). These biomarkers are known to be involved in the regulation of a broad range of biological processes, including DNA double-strand break repair (47). Trimethylated histone H3K9 (H3K9me3) and H3K27me3 are associated with decreased transcriptional activity and heterochromatin condensation (48). On the contrary, histone acetylation, in particular, increased acetylation of the H4 tail, is strongly linked to open, transcriptionally active regions of the chromatin (47).

DNA methylation also regulates microRNA (miRNA) biogenesis (49). miRNAs are small, non-coding RNAs that are recognized as endogenous regulators of post-transcriptional gene expression (5). Under normal physiological conditions, miRNAs function by safeguarding biological processes including cell cycle, proliferation, differentiation, and apoptosis. De-regulation of a single or small subset of miRNAs was reported to have a profound effect on the expression pattern of several hundred mRNAs which propels the cells towards transformation (6), leading to the development and progression of pathological conditions. Both hyper- and hypomethylation of miRNAs represent new levels of complexity in gene regulation (50).

## Epigenetic Changes and Glyphosate-Based Herbicides

Several studies indicate that epigenetic mechanisms could mediate toxicity from pesticides. For example, methoxychlor induced changes in DNA methylation in rat ovary (51) and sperm (52). Moreover, dichlorodiphenyltrichloroethane (DDT) modified DNA methylation in rat hypothalamus (53). Atrazine (ATZ) was reported to dysregulate histone modification in mouse sperm (54, 55) and miRNA levels in rat brain and blood (56).

More recently, epigenetic processes in mammals have been also described after the exposure of glyphosate, GBH, and AMPA. To analyze the published data, we conducted a review of scientific publications on PubMed and Google Scholar searches using the following search terms: “glyphosate” or “GBH” or “AMPA” AND “epigenetic” or “DNA methylation” or “histone modification” or “miRNA” AND “human” or “rat” or “mice”. From these searches, eleven articles were found. Below, these works are described and discussed in detail.

### Glyphosate and AMPA Induced DNA Methylation Changes in Human Cells

Tumor suppressor genes and proto-oncogenes play critical roles in cell cycle regulation, apoptosis, and cell senescence. Moreover, p16, p53, and p21 have important functions in the DNA-damage repair pathways which are among the most frequently compromised pathways in pathological conditions such as tumor growth (57). In this sense, most cancers have inactivating mutations in one or more proteins that normally function to restrict progression through the G_1_ stage of the cell cycle (*e.g.*: p16), and in proteins such as p53 that normally function at crucial cell-cycle checkpoints, stopping the cycle if a previous step has occurred incorrectly or if DNA has been damaged (58). In fact, the hypermethylation of p16 and p53 promoter regions is an epigenetic pattern frequently observed in human cancer development, and this condition is generally associated with reduced methylation level of global genomic DNA (59).

The effect of glyphosate on DNA methylation was first reported by Kwiatkowska et al. (33) *in vitro* (**Table 1**). These authors showed that high concentrations of glyphosate (from 84.54 to 1690 μg/ml) induce DNA lesions in peripheral blood mononuclear cells (PBMCs), decreased global 5mC percentage, and increased methylation of p53 promoter. Recently, similar results were reported, even at lower doses of glyphosate and AMPA (100–1,000 times lesser), in PBMC cells (34, 60) (**Table 1**). Importantly, they found that the hypermethylation of p16, p53, and p21 genes was able to downregulate their mRNA expression and activate proto-oncogenes, which could lead to genomic alterations, downstream function dysregulation, and cancer development risk. Supporting these possible effects, it was later reported by Santovito et al. (61) that human lymphocytes exposed to lower glyphosate concentrations (0.025–0.500 μg/ml) increased the frequency of chromosomal aberration and micronuclei. Later, Woźniak et al. (36) found that glyphosate changes the expression of DNMT1, DMNT3A, and histone deacetylase (HDAC) 3, while AMPA changes the expression of DNMT1 and HDAC3 in PBMCs. These enzymes are involved in the regulation of chromatin architecture and, thus, could affect methylation patterns and histone modification, leading to changes in gene expression. On the other hand, Duforestel et al. (35) found that glyphosate triggered a significant reduction in DNA methylation and increased ten-eleven translocation (TET) 3 activity in MCF10A cells. TET enzymes oxidize 5-methylcytosines and reverse methylation. Combining glyphosate with enhanced expression of miRNA 182-5p (associated with breast cancer) induced tumor development in mice, suggesting that DNA hypomethylation occurring *via* the TET pathway primes cells for oncogenic response in the presence of another potential risk factor (35).

Although controversies have grown about the carcinogenicity and toxicity consequence of glyphosate, effects have been shown on skin cancer promotion in mice and proliferation of human breast cells (62, 63). Taking into account the role of this herbicide as a “probable human carcinogen”, it would be interesting to analyze if the epigenetic changes of tumor suppressor genes observed *in vitro* could be replicated in *in vivo* models and if these molecular alterations could explain, at least in part, some of the adverse effects produce by glyphosate.

### GBH Modifies the Methylation Status of the Estrogen Receptor *α* in Rats

Estrogens, a class of steroid hormones, regulate the growth, development, and physiology of the human reproductive system (64). They are produced principally by the gonads and placenta, but have multiple physiological functions on target organs such as the uterus, hypothalamus, pituitary, bone, mammary tissue, and liver (65). Estrogen signaling is mainly mediated through the classic nuclear receptor, estrogen receptor (ER) *α*. The expression of ER*α* occurs through different promoters depending on the tissue and physiological or developmental stages. In rats, five promoters have been described that result in transcripts with different 5′ untranslated regions derived from exons OS, ON, O, OT, and E1 (66). Importantly, the loss of expression, which is frequently observed in breast cancer, and the presence of triple negative tumors are often associated with hypermethylation of ER regulatory regions (37).

Several works report how the exposure to glyphosate alter the ER expression *in vivo* (67, 68) and *in vitro* (69–72), although the regulation mechanisms involved are still under study. Recently, Gomez et al. (37) and Lorenz et al. (38) found that developmental exposure to GBH (Filial 0, F0) from gestational day (GD) 9 until weaning induces epigenetic changes in ER*α* of F1 rats (**Table 1**). The first group reported that standard diet supplemented with a GBH in two doses, 3.50 and 350 mg of glyphosate/kg bw/day, decreased the expression of OS, O, OT, and E1 transcripts in male mammary glands at postnatal day (PND) 60. These changes were accompanied by an increased in the DNA methylation of their promoter regions (73). Along the same line, Lorenz et al. (38) found, in a similar model, that perinatal exposure to GBH in a dose of 350 mg of glyphosate/kg bw/day, upregulated the expression of ER*α* mRNA in the pregnant rat uterus (F1) at GD5 (preimplantation period). This change was associated with an increase in the abundance of the O transcript variant and a decrease in DNA methylation of its promoter. Supporting these transcriptional changes, histone H4 acetylation and H3K9me3 were enriched in the O promoter in GBH-exposed rats, whereas H3K27me3 was decreased.

These studies proposed that the adverse observed effects of GBH on mammary gland growth (37, 73–75), embryo implantation (76), uterine development and reproduction (67, 68, 77), could be mediated, at least in part, by aberrant DNA methylation and histone acetylation/methylation of ER*α* gene. In this sense, the disruption of ER was previously related to male and female outcomes, including infertility, abnormal uterine and sperm maturation, atypical ovarian functions, and implantation deficits (78). These results require particular attention since all the epigenetic alterations mentioned above were observed after the GBH exposure has ended, suggesting that this exposure during sensitive periods of development (gestation) could perturb epigenetic programming and could have a long-lasting impact later in life. So far, it is necessary to clarify whether these effects could be due to the active principle (glyphosate), the co-formulants, or a combination of both, since previous studies have shown that commercial formulations are more toxic than glyphosate alone (79). In addition, it would be interesting to consider different administration routes, timings of exposure, and time points as conditioning factors.

### Glyphosate Induced Epigenetic Transgenerational Inheritance

Epigenetic transgenerational inheritance is a non-genetic form of inheritance that allows environmental factors to produce epigenetic alterations in the germline (sperm or egg) at critical periods of development that could be passed to subsequent generations, leading to pathologies or phenotypic variation in the absence of continued direct exposures (41). DNA methylation reprogramming could occur in the early embryo following fertilization (80), in the primordial germ cells in early gonadal development (81) or, even, during adult spermatogenesis in the testis (1).

Recently, Deepika Kubsad et al. (41) studied the transgenerational effect of glyphosate exposure (25 mg/kg bw/day) on pregnant rats (F0) during GD8 to 14. They found that this exposure produced in F1, F2, and F3 differential DNA methylation regions (DMRs) in the sperm (**Table 1**). DMR associated gene categories were mainly related to transcription, signaling, metabolism, receptors, and cytokines and include metabolic and cancer pathways. Negligible pathology was observed in the F0 and F1 generations, while a significant increase in prostate, kidney and ovarian diseases, obesity, and parturition (birth) abnormalities was observed in the F2 generation grand-offspring and F3 generation great-grand-offspring. Tumor development was also monitored in males and females and found to increase in the F2 generation glyphosate female lineage; the most predominant were mammary adenomas. In another work from the same group, sperm from F3 generation was studied for DMRs and differential histone retention sites (DHRs), that were correlated with known pathology specific-associated genes (42) (**Table 1**). Interestingly, overlapping sets of DMRs and DHRs were identified that were common for all the pathologies. These results support previous works that found adverse effects related to fetal parameters and structural congenital anomalies after perinatal exposure of GBH (F0) in second-generation of rats (F2) (76, 82, 83). However, Deepika Kubsad et al. (41) and Maamar et al. (42) reported for the first time that transgenerational inheritance of disease in rodents could be produced by DMRs and DHRs in the male germline, and these sites could potentially act as a biomarkers for specific diseases. Based on these findings, further studies are needed to deepen on the generational toxicology of glyphosate, in the disease etiology of the future generations.

### Maternal Exposure to GBH Alter the Non-Coding RNAs Profile in the Rat Offspring

The effect of GBH on miRNAs was recently reported by Hua ji et al. (39) who studied the effect of glyphosate exposure (1% Roundup; equivalent to 50 mg/kg bw/day) during pregnancy and lactation (GD14 to PND7) in the offspring brain (**Table 1**). A miRNA microarray detected 55 upregulated (*i.e*: miR-711, miR-27b-3p, miR-142a-3p) and 19 downregulated (*i.e*: miR-34b-5p) miRNAs in the prefrontal cortex of mice at PND28 after maternal exposure. In addition, they reported abnormalities of the Wnt/*β*-catenin and Notch pathways in these animals that correspond with the dysregulation found in miRNA patterns. This support previous works that showed a disruption of Wnt proteins by neonatal exposure to GBH (2 mg/kg bw/day) from PND1 to PND7, in rat uterus (PND21) and implantation sites (GD9) (68, 77). In addition, exposure to glyphosate also produced a downregulation of these pathways in neuron cultures (84). In a second work and using the same model of exposure, these authors also found that circular RNA (circRNA) profile was significantly altered in the hippocampus of perinatal glyphosate exposure group (40) (**Table 1**). circRNAs are a special class of non-coding RNAs which may interact with miRNAs to regulate gene expression. The altered miRNA and circRNAs were related to biological functions, including neurogenesis, neuron differentiation, brain development, stress-associated steroid metabolism pathways, among others.

Some of the miRNAs and their target genes disrupted by glyphosate exposure were also reported to be involved in pathological conditions, such as neurological disorders, prostate cancer and breast cancer (40, 85–88). Particularly, miR-34b-5p affects Numbl* *and Notch1 genes, which are involved in the Notch signaling pathway. In addition, the 3′-untranslated regions of *β*-catenin and Lef-1, which are involved in the Wnt signaling, contain miR-34 binding sites and are sensitive to miR-34b-dependent regulation. Abnormal activation of the Wnt/*β*-catenin or Notch pathways may serve an important role in the pathogenesis of various reproductive outcomes, including preeclampsia (85), embryo implantation (86), endometriosis (87), and ovarian tumors (88). Taking all together, these findings provide a new basis for identifying the mechanism of action of glyphosate-induced neurotoxicity in the developing brain and could serve as a beginning for elucidating the more general mechanisms of GBH toxicity in human and animal models. In addition, more investigations are needed to clarify the interaction between circRNAs, miRNAs, and genes as possible target of glyphosate exposure.

## Conclusions and Future Perspectives

**Figure 1:**
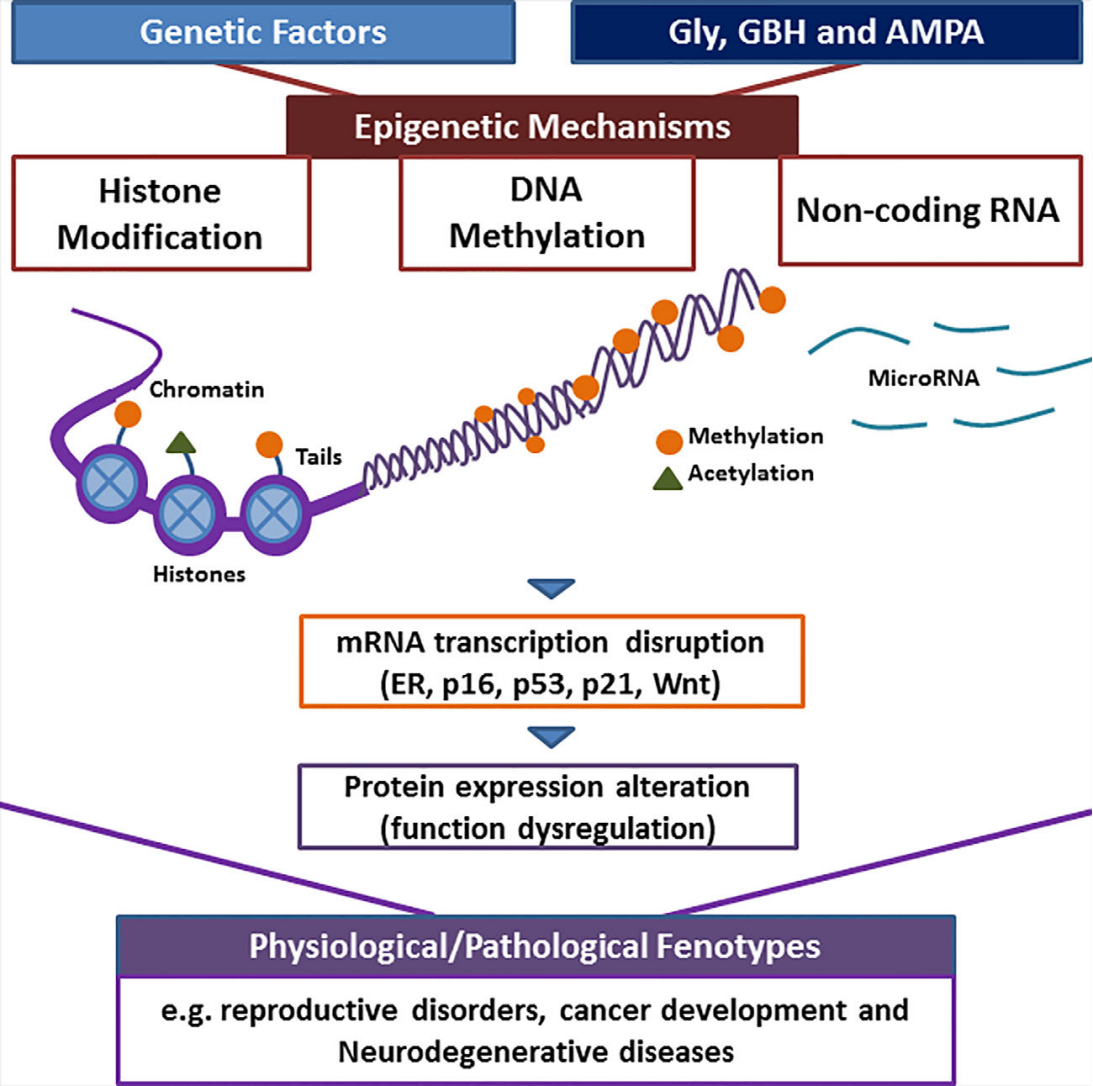
Possible mechanism that links the exposure to herbicides, the epigenome modification and the observed phenotypes. Chromosomes are composed of chromatin wrapped around proteins called histone; modifications of histone tails and DNA methylation control transcriptional access to DNA. Non-coding RNAs also regulate transcription. The exposure of herbicides, such as glyposhate (Gly), glyphosate-based herbicides (GBH) and, its main metabolite, (aminomethyl) phosphonic acid (AMPA) could alter the epigenome and could produce the silencing/activating of numerous genes, including estrogen receptor (ER), p16, p21, p53, and Wnt. This could result in the disruption of physiological functions and the promotion of health outcomes.

Recent findings have shown that the exposure of glyphosate, GBH, or AMPA could affect epigenetic mechanisms. These include the decrease of global DNA methylation, alterations in the methylation pattern of specific regions, including ER and tumor suppressor genes, histone modifications, and differential expression of non-coding RNAs involved in, for example, Wnt and Notch pathways. These epigenetic markers have been involved in several physiological and pathological processes that were also reported after glyphosate, GBH, or AMPA exposure in animal models. In this sense, several lines of evidence indicate that the exposure to these compounds could alter the epigenome, disrupting the mRNA expression and protein levels of key genes involved in normal functions and thus, producing negative consequences (**Figure 1**). These epigenetic alterations could be heritable and could have a manifestation in health impacts and disease after the exposure has ended. Overall, more studies are needed to identify epigenetic targets, to define how they are dysregulated in human disease and their functional role, and to determine the critical windows of vulnerability by herbicide exposures. These points would influence environmental risk assessment and contribute to the development of prevention strategies for health outcomes.

## Author’s Note

MR, GC, and VL are fellows, and MM, JV, and JR are Career Investigators of the Consejo Nacional de Investigaciones Científicas y Técnicas (CONICET), Argentina.

## Author Contributions

MR, GC, VL, MM, JV, and JR contributed equally to the literature search and to manuscript writing, revising, and editing. All authors contributed to the article and approved the submitted version.

## Conflict of Interest

The authors declare that there is no conflict of interest that could be perceived as prejudicing the impartiality of the research reported.

## Abbreviations

5mC, 5-methylcytosine; AMPA, (aminomethyl)phosphonic acid; ATZ, atrazine; circRNAs, circular RNAs; DNMT, DNA methyltransferase*;* EFSA, European Food Safety Authority; EPA, Environmental Protection Agency; ER, estrogen receptor; F, Filial; GBHs, glyphosate-based herbicides; GD, gestational day; HDAC, histone deacetylase; miRNA, microRNA; PBMC, peripheral blood mononuclear cell; PND, postnatal day.
